# Detailed assessment of axial and peripheral entheses and joints in axial spondyloarthritis and psoriatic arthritis patients treated with ixekizumab (DAPHNE): design of a 2-year phase IV trial applying whole-body MRI, MRI-based synthetic CT, and CT

**DOI:** 10.1186/s41927-025-00560-5

**Published:** 2025-10-16

**Authors:** Simone Tromborg Willesen, Jakob Møllenbach Møller, Susanne Juhl Pedersen, Mikkel Østergaard

**Affiliations:** 1 Copenhagen Center for Arthritis Research, Center for Rheumatology and Spine Diseases, Rigshospitalet, Glostrup, Denmark; 2 Department of Clinical Medicine, Faculty of Health and Medical Sciences, University of Copenhagen, Copenhagen, Denmark; 3 Department of Radiology, Copenhagen University Hospital at Herlev Gentofte, Copenhagen, Denmark

**Keywords:** Axial spondyloarthritis, Psoriatic arthritis, Magnetic resonance imaging, Computed tomography

## Abstract

**Background:**

Ixekizumab, an interleukin 17A inhibitor, has demonstrated efficacy in improving clinical and patient-reported outcomes in axial spondyloarthritis (axSpA) and psoriatic arthritis (PsA). However, objective data regarding its effects on peripheral inflammation in joints and entheses, inflammation of the posterolateral spinal segments, and structural progression in the spine are lacking. In this study, we aim to address the abovementioned gaps by conducting a longitudinal investigation of the effects of ixekizumab on peripheral and axial inflammation and structural damage in patients with axSpA and PsA. Through comprehensive assessment using advanced imaging techniques such as whole-body magnetic resonance (WB-MRI) and detailed MRI evaluation of the spine, including MRI-based synthetic computed tomography (synthetic CT), and low-dose CT, we seek to investigate the therapeutic effectiveness of ixekizumab across different disease domains.

**Methods:**

DAPHNE (EU-CT 2024-510746-14–00) is a 2-year open-label investigator-initiated multi-center study conducted in Denmark. Sixty-five patients with axSpA and PsA (≥25 with each diagnosis; ≥10 PsA patients with imaging-documented axial involvement), with a clinical indication for biological disease-modifying antirheumatic drug therapy, will be treated with ixekizumab, and followed by clinical, laboratory, WB-MRI, radiography, and low dose CT of sacroiliac joints (SIJs) and spine. MRI will include T1-weighted and short tau inversion recovery (STIR) sequences and a sequence allowing the generation of CT-like “synthetic CT” MR images. Images will be evaluated by readers blinded for diagnosis and clinical and other imaging findings. Furthermore, new lesion definitions and evaluation methods for synthetic CT and low-dose CT will be sought developed and explored.

**Discussion:**

Currently, there are no data on the objective assessment of enthesitis, inflammation of the posterolateral segments of the spine, or structural progression in the spine using modern imaging during ixekizumab treatment neither in PsA nor axSpA. Thus, a longitudinal study hereof - the DAPHNE study - is anticipated to significantly improve our understanding of the diverse disease manifestations in axSpA and PsA, and how they respond to ixekizumab treatment. Additionally, the study will provide valuable insights into new and improved imaging examination and evaluation methods.

**Clinical trial number:**

EU-CT 2024-510746-14-00.

## Introduction

Spondyloarthritis (SpA) comprises a group of inflammatory rheumatic diseases, including axial spondyloarthritis (axSpA) and psoriatic arthritis (PsA), characterized by inflammation affecting axial and peripheral joints as well as entheses [1]. The diverse manifestations of SpA cause pain, fatigue, and functional impairment [2–4]. Nevertheless, conventional clinical and radiographic assessments often lack sensitivity and specificity, particularly in diagnosing enthesitis, a hallmark pathology in PsA and axSpA [5].

Ixekizumab, an interleukin 17A inhibitor, has demonstrated efficacy in improving clinical and patient-reported outcomes in axSpA and PsA [6–9]. However, objective data regarding its effects on peripheral inflammation in joints and, most importantly, entheses is lacking.

Imaging modalities such as ultrasonography and magnetic resonance imaging (MRI) can visualize inflammation in peripheral entheses and joints [10]. Whole-body MRI (WB-MRI) presents a comprehensive approach to assess both peripheral and axial manifestations in a single examination [11, 12]. Validated assessment methods for WB-MRI in SpA patients, as the OMERACT MRI Whole-Body Score for Inflammation in Peripheral Joints and Entheses (MRI-WIPE) [13, 14], provide valuable tools for objective evaluation in addition to differentiating PsA and axSpA manifestations and their response to therapy.

Patients with axSpA and axial PsA demonstrate inflammation and damage at various anatomical locations in the spine on MRI, including the vertebral bodies and the “posterior segments” of the spine, i.e. the costo-transversal joints, costo-vertebral joints, facet joints, spinous/transverse processes, and the surrounding soft tissue [15, 16]. While methods like the Spondyloarthritis Research Consortium of Canada (SPARCC) MRI Spine index and the Berlin modifications of the ASspiMRI-a have been validated and are often used in clinical trials [17, 18], they do not capture inflammation in posterior segments of the spine or in general provide detailed anatomical information as they do not record where in the vertebral bodies the inflammation is located. The Canada-Denmark (CANDEN) MRI assessment method for spinal lesions offers a more comprehensive approach, enabling detailed evaluation of various anatomical locations within the spine, including the posterior segments of the spine [19, 20]. Currently, there are no data regarding the effectiveness of ixekizumab on spinal inflammation as assessed by this detailed method.

Assessing structural progression in the spine in patients with SpA, which is crucial for determining a therapy’s structure-modifying effects, remains a challenge. Conventional radiography using the modified Stoke Ankylosing Spondylitis Spinal Score (mSASSS) [21] lacks sensitivity to change [22] and cannot adequately assess structural damage progression in short-term trials (≤2 years) [23, 24]. Computed tomography (CT) offers superior visualization of structural damage [25] but is limited by radiation exposure. A promising alternative is MRI-based synthetic CT (sCT) [26], which provides CT-like images without radiation exposure. However, its utility in assessing structural damage progression in the spine in patients with axSpA and PsA remains unexplored.

In this study, we aim to address the abovementioned gaps by conducting a longitudinal investigation of the effects of ixekizumab on peripheral and axial inflammation and structural damage in patients with axSpA and PsA. Through comprehensive assessment using advanced imaging techniques such as WB-MRI and detailed MRI evaluation of the spine, including sCT, and low-dose CT, we seek to provide valuable information on the therapeutic effectiveness of ixekizumab across different disease domains. The primary objective is to assess the effect of ixekizumab on the total inflammatory burden in the entire body (peripheral and axial joints and entheses) of patients with axSpA and PsA, as assessed by WB-MRI.

Here we report the design of the DAPHNE study.

## Methods/Design

### Study design

**Fig. 1:**
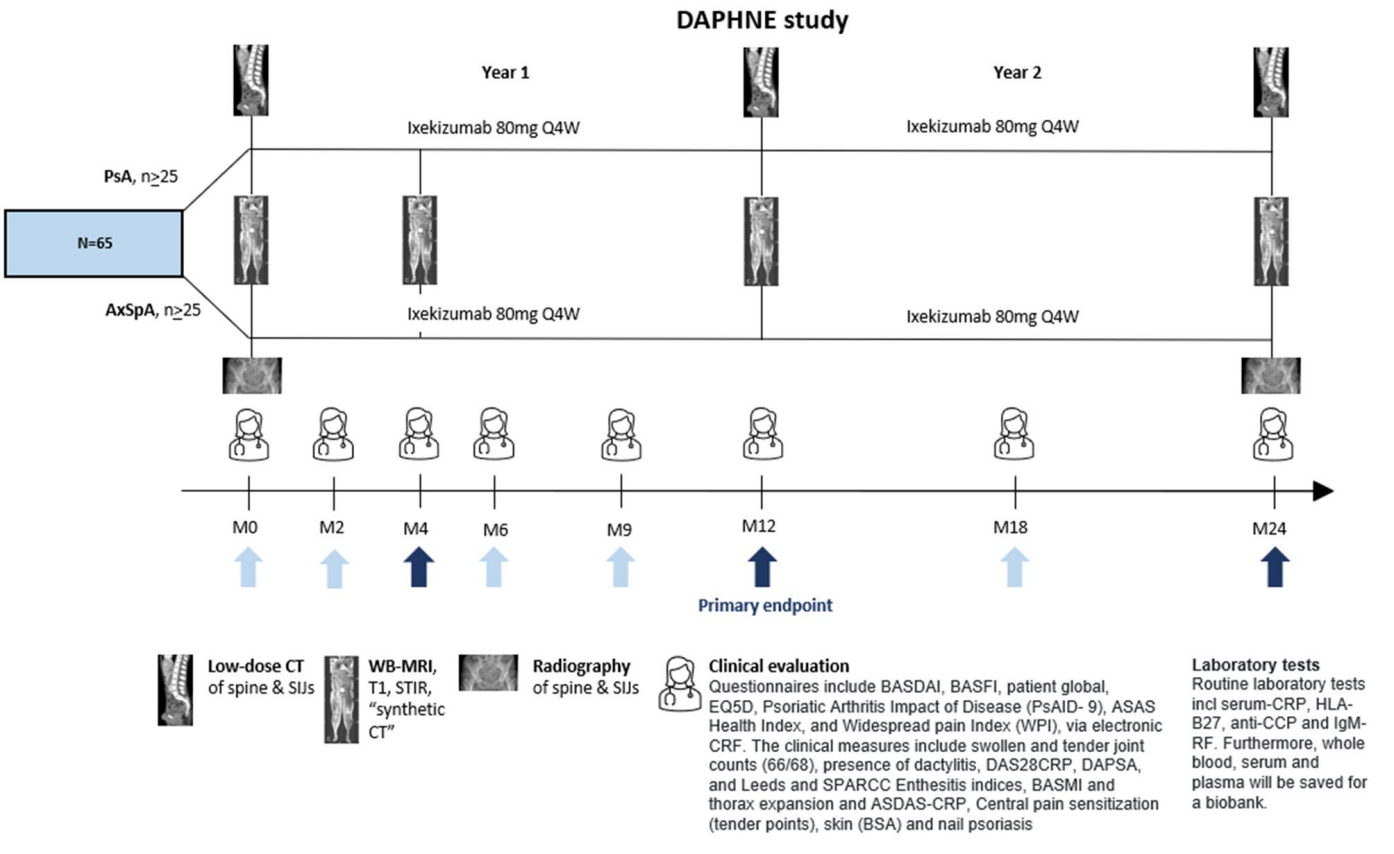
Design of the DAPHNE study. *PsA *psoriatic arthritis, *AxSpa* axial spondyloarthritis, *CT* computed tomography, *SIJs* sacroiliac joints, *WB-MRI* whole Body magnetic resonance imaging, *STIR* short tau inversion recovery, *synthetic CT* MRI-based synthetic computed tomography, *BASDAI* the bath ankylosing spondylitis disease activity index, *BASFI* the bath ankylosing spondylitis functional index, *ASAS* assessment in SpondyloArthritis International society, *DAS* disease activity index, *DAPSA* disease activity index for psoriatic arthritis, *BASMI* the bath ankylosing spondylitis metrology index, *ASDAS* ankylosing spondylitis disease activity score, *CRP* C-reactive protein, *BSA* Body surface area, *HLA-B27*, human leukocyte antigen B27, *anti-CCP* anti-citrullinated protein, *RF* rheumatoid factor. Dark arrows: Major assessment visits

DAPHNE (EU-CT 2024 -510746-14–00) is a 2-year open-label investigator-initiated multi-center study conducted in Denmark. Sixty-five patients with axSpA and PsA (≥25 with each diagnosis; ≥ 10 PsA patients with imaging-documented axial involvement), with a clinical indication for biological disease-modifying antirheumatic drug (DMARD) therapy, will be treated with ixekizumab, and followed by clinical and laboratory examination (months 0, 2, 4, 6, 9, 12, 18, 24), WB-MRI (months 0, 4, 12 and 24), radiography (months 0 and 24) and low dose CT of sacroiliac joints (SIJs) and spine (months 0, 12 and 24) (see Fig. 1). MRI will include T1-weighted and short tau inversion recovery (STIR) MRI and a sequence allowing the generation of CT-like “synthetic CT” MR images.

### Study interventions

All patients (axSpA and PsA) will be treated with ixekizumab. The study medicine will be provided as prefilled pens with ixekizumab 80 mg dispensed in 1 ml sterile water. At week 0 (baseline) the patients are treated with subcutaneous (sc.) ixekizumab 160 mg (2 injections of 80 mg), thereafter 80 mg at week 2 and week 4. From week 4 and throughout the study the patients are treated with sc. ixekizumab 80 mg every 4 weeks. The first sc. injections of ixekizumab will be given at the participating centers under the supervision of a study nurse. Hereafter, the patient will be allowed to self-administer the study medicine at home.

Patients are allowed to continue the following concomitant medications: conventional synthetic (cs) DMARDs (dose must be fixed from 1 month before the first MRI scan to month 4), non-steroid antirheumatic drugs (NSAID) (dose must be fixed from 1 month before the first MRI scan to month 4), and paracetamol. Injections of glucocorticoids in peripheral joints and/or entheses should preferably be avoided and are only allowed at specific time points during the study; these periods are before 1 month before the first MRI scan, from month 4 (after all study procedures have been performed) to month 11, and from month 12 (after all study procedures have been performed) to month 23.

Other drugs with a potential disease-modifying effect (except csDMARDS) and high-potency opioid analgesics for musculoskeletal pain are prohibited during the study.

### Participants

Participants will be recruited from rheumatological outpatient clinics in the capital region of Copenhagen. Participants in this study are all patients followed by the participating rheumatologic departments. The participant must have moderate to high disease activity, and it must have been decided by the individual departments (at a conference between rheumatologists), that the patient should start or switch biologic treatment.

The patient is invited to participate in the study by a physician in the rheumatological outpatient clinic during a routine visit. Oral and written information will be provided. If the patient agrees to participate, an informed consent form is signed. A screening visit is performed to ensure that the patient meets the criteria for participation.

Inclusion criteria for both groups (axSpA and PsA) are age $\ge$ 18 years at the time of consent, a clinical indication for a biological drug as assessed by the treating physician, sufficient contraception for women in addition to the ability and willingness to provide written informed consent and compliance with the requirements of the study protocol.

For patients with axSpA additional inclusion criteria are a diagnosis of axSpA according to the Assessment of SpondyloArthritis International Society (ASAS) classification criteria [27, 28], active inflammation on MRI of the SIJs and/or spine [29] as evaluated by a central SpA imaging expert and/or radiographic modified New York criteria [30] fulfilled, and total back pain as measured on a VAS scale ≥40 mm (0–100 mm) at baseline.

For patients with PsA additional inclusion criteria are PsA according to Classification Criteria for Psoriatic Arthritis (CASPAR) [31], negative rheumatoid factor (RF) and anti-cyclic citrullinated peptide (anti-CCP), and ≥ 1 tender and swollen joint in addition to two further anatomical localizations with clinical signs of inflammation (i.e., tender enthesis and/or tender and/or swollen joint) assessed at screening and baseline. For patients to fulfill the axial PsA component they must have imaging documented axial involvement on MRI, radiography, or low-dose CT as judged by a central SpA imaging expert.

Exclusion criteria includes contraindications to ixekizumab (serious hypersensitivity to the active substance or the excipients, clinically important active infections (e.g., active tuberculosis), and administration of live vaccines during treatment with ixekizumab) or to MRI (such as metal implants, pregnancy, pacemaker or other implants or electronic devices incompatible with MRI); prior exposure to ixekizumab or other biologic agents targeting IL-17 or the IL-17 receptor; treatment with more than one prior TNF inhibitor; and prior use of bDMARDs other than TNF inhibitors. Patients with active inflammatory bowel disease are also excluded. Use of any investigational drug or device within one month prior to randomization, or within five half-lives of the investigational product (whichever is longer), is not permitted. Additionally, any use of oral, intravenous, intra-articular, or intramuscular corticosteroids within one month prior to the baseline MRI is grounds for exclusion. DMARDs are permitted during the study, provided the dose remained stable for at least one month prior to the first MRI. Further exclusion criteria includes pregnancy or lactation; known recent drug or alcohol abuse; diagnosis of a severe psychiatric disorder; inability to comply with study procedures due to physical or mental limitations; and inability to speak, read, or understand Danish.

### Assessments

During the 2-year study period, patients will have eight visits to the outpatient clinic, three of which are major assessment visits (months 4, 12, and 24) (see Fig. 1, dark blue arrows). At the major assessment visits, treatment efficacy, and disease activity are evaluated. Patients with ASDAS low disease activity (ASDAS ≤2.1) (axSpA) or DAPSA low disease activity (DAPSA ≤14) (PsA) continue treatment with sc. inj. ixekizumab 80 mg every 4 weeks. Patients, who are not responding (defined as having ASDAS > 2.1 or DAPSA > 14), change treatment at the discretion of the treating rheumatologist and according to the available guidelines from the Medicine Council, Danish Regions (Medicinrådet, Danske Regioner), and they continue to be followed in the study.

All protocol-related assessments will be entered into an electronic case report dedicated to the DAPHNE study.

#### Magnetic resonance imaging (MRI)

Whole-body MRI, including conventional MRI (T1 weighted and STIR sequences) and an MRI sequence used for synthetic CT reconstruction (3D gradient echo sequences), will be performed on all patients at baseline, month 4, month 12, and month 24.

WB-MRI will be performed with patients in a supine position and for the following areas: 1. coronal images of shoulders and anterior chest wall; 2. sagittal images of the cervical, thoracic, and lumbar spine; 3. coronal images of the pelvis and hips; 4. coronal images of hands (positioned under the buttocks); 5. sagittal images of knees; 6. sagittal images of ankles; and 7. axial images of the ankles/feet. 3 mm slice thickness (ST) will be used for hand, wrist, and forefeet (in-plane resolution 0.6x0.6 mm), 4 mm ST for spine and SIJ, and 5 mm ST for anterior chest wall, shoulders, pelvis, knee, and ankles (in-plane resolution 0.5x0.5 mm).

The MRI scans will be performed on a 3 Tesla MRI unit.

#### MRI-based synthetic CT

BoneMRI or “synthetic CT” is a quantitative 3D MRI technique, developed by the company MRIGuidance BV, NL, based on a multiple gradient-echo sequence (TR, TE, FlipAngle of 7 ms, 2 ms, and 3.5 ms and 10°; ST 0.8 and in-plane resolution 0.6x0.6 mm) and a machine learning processing pipeline [32]. The BoneMRI technology can generate CT-like, quantitative radiodensity bone MRI images to visualize cortical and trabecular bone, allowing assessment of bone structure and morphology.

The bone MRI sequences will be reconstructed by MRIGuidance BV, NL, using the BoneMRI model. MRIGuidance BV, NL will have no access to other MR images, CT images, or radiographs during the study.

#### Computed tomography

Low-dose CT of the spine (cervical, thoracic, and lumbar spine) and SIJs will be performed at baseline and repeated at months 12 and 24.

The low-dose CT is performed in a photon-counting CT scanner using a dedicated low-dose protocol. The kilovoltage peak is set at Sn100 kVp, and automated tube current modulation is used. The scan field of view is 50 cm. Quantum iteration level 3 is used, the pitch is 0.5, collimation 144 × 0.4, and rotation time 0.5 s. The requested CT-dose index is 1.36 mGy. The total examination time is approximately 10 minutes. The axial images are reconstructed in sagittal and coronal planes for the spine and coronal- and axial-oblique planes for the SIJs.

#### Radiography

Radiographs of the SIJs and spine (cervical and lumbar spine) will be performed at baseline and repeated at month 24. The projections will be performed with the patient in a standing position in accordance with the mSASSS recommendations [33], and include anterior-posterior pelvis, and lateral cervical and lumbar spine projections. The total examination time is approximately 15 minutes.

#### Image evaluation

Image evaluation will be by state-of-the-art internationally validated methods for radiographs (modified New York criteria [30] and the mSASSS [33] method), MRI (SPARCC sacroiliac inflammation [17] and structural (SSS) [34] scores, Canada-Denmark spine score of inflammation and damage [19, 20]), and OMERACT Whole-body MRI scores of peripheral enthesitis and joint inflammation scores (WIPE) [13, 14]. Images will be evaluated by readers blinded for diagnosis and clinical and other imaging findings. Furthermore, new lesion definitions and evaluation methods for synthetic CT and low-dose CT will be sought developed and explored.

#### Other assessments

Details of other assessments (clinical, composite, and patient-reported assessments) are provided in Table 1 and a summary of all measures to be collected is provided in Table 2.

**Table 1:** Details of clinical, composite and patient-reported assessments

Clinical evaluation and composite indices	
Inflammatory back pain (ASAS definition)	Criteria set for inflammatory back pain according to ASAS experts include age at onset < 40 years, insidious onset, improvement with exercise, no improvement with rest, and pain at night [35].
ASDAS CRP	A composite measure of disease activity based on CRP, VAS of patient global assessment of disease activity (0–100 mm), back pain (0–100 mm), duration of morning stiffness (0–100 mm), and peripheral pain/swelling (0–100 mm) [36].
BASMI	Five measurements of different movements are performed, including intermalleolar distance, flexion and lateral flexion of the lumbar spine, tragus-wall distance, and rotation of the neck [37].
DAS28 CRP	An index based on tender and swollen joint count of 28 peripheral joints, patient global assessment of disease activity, and CRP [38].
CDAI	CDAI is a composite index based on tender and swollen joint count and patient and physician global assessment of disease activity on VAS [39].
Swollen and tender joint count	In total 66 joints are examined for swelling and 68 for tenderness in all patients. The following joints are examined: right and left temporomandibular joint, sternoclavicular joint, acromioclavicular joint, glenohumeral articulation, elbow joint, wrist, 10 MCP joints, 10 PIP- and DIP joints in the hands and 10 toes and MTP-joints, tarsus/midfoot joint, ankle joint, knee joint, hip joint (for tenderness only).
SPARCC Enthesitis Index	SPARCC Enthesitis index comprises 18 anatomical sites, nine on the right side and nine on the left side: Insertion of supraspinatus, lateral and medial epicondyle humerus, greater trochanter, quadriceps insertion into the superior border of patella, patellar tendon insertion into the inferior pole of patella or tibial tubercle, Achilles tendon and insertion plantar fascia, with a score of 0–16 (for scoring purpose, the inferior patella and tibial tuberosity are considered one site because of their anatomical proximity) [40].
Leeds Enthesitis Index	LEI comprises six anatomical sites: The lateral epicondyle of humerus (left/right), the medial epicondyle of femur (left/right), and the Achilles tendon (left/right), with a score of 0–6 [41].
DAPSA	An index based on swollen and tender joint counts, patient global and pain assessment, and CRP [42]
Minimal Disease Activity (MDA)	A state of MDA for PsA has been established and is defined by a low activity of disease assessed by tender/swollen joint counts, tender entheseal points, Psoriasis Area and Severity Index or body surface area, patient pain and global activity VAS, and functional evaluation by Health Assessment Questionnaire (HAQ) [42].
Physician global assessment	The physician evaluates the overall disease activity of the patient (VAS 0–100 mm).
Extraarticular SpA features	Evaluation of presence of symptoms/signs of activity in the following extraarticular SpA features: anterior uveitis or scleritis, inflammatory bowel disease, dactylitis, skin or nail psoriasis, and heel enthesitis.
Tender points	Tender points are defined by the ACR as 18 points on the body, nine on one side, and nine on the other, where pain can be felt immediately beneath the skin when pressed. According to the 1990 ACR guidelines, fibromyalgia could be diagnosed based on the presence of tender points [43].
BSA	BSA assessment measures the total area of the body affected by psoriasis. Psoriasis that occurs in less than 5% of the BSA is considered mild, 5–10% is moderate and if psoriasis affects more than 10% of the BSA, it is severe. Hand surface area (HSA) or palm surface area (PSA) is commonly used for the estimate, with an assumption that HAS represents 1% and PSA (approximately equal to the palm of the patient’s hand, excluding fingers) represents 0,5% of BSA in adults [44].
Nail involvement, absence/presence of psoriasis	Nail involvement will be evaluated for psoriatic changes such as onycholysis, pitting, and hyperkeratosis with a count of the number and location of affected nails (presence/absence of psoriatic change) [42].
**Patient reported outcomes**	
Patient global score, pain, and fatigue	The patient’s general assessment of the influence of disease on global health, pain, and fatigue is indicated on a VAS [45, 46].
BASDAI	The patient indicates the severity of six types of discomfort related to disease activity on six individual VAS, including the degree of back pain. A BASDAI index is hereafter calculated based on the patient’s replies to the questions [47].
BASFI	The patient indicates the ability to perform ten actions or movements on a VAS. The questions are primarily related to axial arthritis. A BASFI index is hereafter calculated based on the patient’s replies to the questions [48].
HAQ	The patient’s functional status is assessed using a questionnaire with 20 questions that evaluates how difficult it is for the patient to perform normal daily activities divided into eight different categories. The questions are primarily related to peripheral joints. The degree of overall pain on a VAS is included. A HAQ score is calculated. The questionnaire is extended to include five additional questions (two categories) in relation to axSpA [49].
EQ-5D	The patient’s assessment of the health-related quality of life (HRQoL) with EQ5D comprises five questions about five different domains of HRQoL and a VAS on the patient’s perception of his/her/their health [50].
ASAS Health Index	ASAS Health Index is a questionnaire that measures functioning and health across 17 aspects of health and nine environmental factors in patients with SpA [51].
PASS	PASS is defined as the highest level of symptom beyond which patients consider themselves well. A question about disease status as assessed by the patient at the current visit with the last visit as an anchor, and whether the patient finds the current state of disease activity acceptable (yes/no) [52].
PsAID-9	The PsAID Questionnaire is a patient-reported outcome measure of disease impact in PsA and its domains are entirely patient-generated [53].
WPI	The WPI quantifies the extent of bodily pain on a 0–19 scale by asking patients if they have had pain or tenderness in 19 different body regions (shoulder girdle, hip, jaw, upper arm, upper leg, lower arm, and lower leg on each side of the body, as well as upper back, lower back, chest, neck, and abdomen) over the past week, with each painful or tender region scoring 1 point [54].
Injection Site Questionnaire	A questionnaire that was developed for this study uncovering the overall injection experience with Taltz. Patients mark the box that best fits their injection experience (very satisfied, somewhat satisfied, neither satisfied nor dissatisfied, somewhat dissatisfied, very dissatisfied)

*ASAS* assessment in SpondyloArthritis International Society, *ASDAS* Ankylosing Spondylitis Disease Activity Score, *CRP* C-reactive protein, *VAS* Visual analog scale*, BASMI* The Bath Ankylosing Spondylitis Metrology Index, *DAS* Disease Activity Index, *DAPSA* Disease Activity Index for Psoriatic Arthritis, *CDAI* Clinical Disease Activity Index, *MCP* Metacarpophalangeal, *PIP* Proximal interphalangeal, *DIP* Distal interphalangeal, *MTP* Metatarsophalangeal, *SPARCC* The Spondyloarthritis Research Consortium of Canada, *SpA* Spondyloarthritis, *ACR* American College of Rheumatology, *BSA* Body Surface Area, *BASDAI* The Bath Ankylosing Spondylitis Disease Activity Index, *BASFI* The Bath Ankylosing Spondylitis Functional Index, *HAQ* Health Assessment Questionnaire, *PASS* Patient Acceptable Symptom State, *PsAID* Psoriatic Arthritis Impact of Disease, *WPI* Widespread Pain Index

**Table 2:** Summary of measures to be collected

Visit number	S	1	2	3	4	5	6	7	8
Month*	− 1/2	0	2	4	6	9	12	18	24
**Inclusion related procedures**									
Informed consent	X								
Clinical history	X								
Past medical & surgical history	X								
Inclusion & exclusion criteria	X	X							
**Background factors**									
Gender & age	X								
Diagnosis (axSpA, PsA)	X								
Symptom duration & year of diagnosis	X								
Tobacco	X								
Height (cm) & weight (kg)	X								
**Patient-reported outcomes**									
Patient global score, pain & fatigue	X	X	X	X	X	X	X	X	X
BASDAI, BASFI, HAQ, EQ-5D, ASAS Health Index	X	X	X	X	X	X	X	X	X
PASS, PsAID-9, WPI	X	X	X	X	X	X	X	X	X
Injection site questionnaire			X						
**Clinical evaluation & composite indices**									
ASDAS CRP	X	X		X			X		X
Inflammatory back pain	X	X		X			X		X
BASMI	X	X		X			X		X
DAS28 CRP, CDAI, DAPSA, MDA	X	X		X			X		X
Swollen and tender joint count	X	X		X			X		X
Enthesitis (SPARCC and Leeds)	X	X		X			X		X
Tender points	X	X		X			X		X
Physician global (VAS 0-100 mm)	X	X		X			X		X
Extraarticular SpA features **	X	X		X			X		X
Physical examination*** incl. blood pressure & pulse	X	X		(X)			(X)		(X)
**Evaluation of treatment effect**									
ASDAS Low disease activity (axSpA)				X			X		X
DAPSA Low disease activity (PsA)				X			X		X
**Imaging**									
WB-MRI (incl. Synthetic CT)	X			X			X		X
Low-dose CT of SIJs & spine	X						X		X
Radiography of SIJs & spine	X								X
Radiography of thorax	X								
**Laboratory procedures**									
HLA-B27, anti-CCP & RF	X								
hCG (serum or urine, women only)	X	(X)	(X)	(X)	(X)	(X)	(X)	(X)	(X)
Screening blood tests**** before “biologics” incl. ANA	X								
Routine blood tests***** incl. CRP	X	X	X	X	X	X	X	X	X
Blood samples stored for biobank		X		X			X		X

X = assessed, (X) = assessment optional. *axSpA* axial spondyloarthritis, *PsA* Psoriatic Arthritis, *BASDAI* The Bath Ankylosing Spondylitis Disease Activity Index, *BASFI* The Bath Ankylosing Spondylitis Functional Index, *HAQ* Health Assessment Questionnaire, *ASAS* assessment in SpondyloArthritis International Society, *PASS* Patient Acceptable Symptom State, *PsAID* Psoriatic Arthritis Impact of Disease, *WPI* Widespread Pain Index, *ASDAS* Ankylosing Spondylitis Disease Activity Score, *CRP* C-reactive protein, *BASMI* The Bath Ankylosing Spondylitis Metrology Index, *DAS* Disease Activity Index, *CDAI* Clinical Disease Activity Index, *DAPSA* Disease Activity Index for Psoriatic Arthritis, *MDA* Minimal Disease Activity, *VAS* Visual analog scale, *SpA* Spondyloarthritis, *WB-MRI* Whole-body magnetic resonance imaging, *CT* computed tomography, *HLA-B27*, human leukocyte antigen B27, *anti-CCP* anti-citrullinated protein, *RF* rheumatoid factor

*A month is defined as 30 days

**Extraarticular SpA features include presence of symptoms/signs of activity in the following: anterior uveitis or scleritis, inflammatory bowel disease, dactylitis, skin or nail psoriasis, and heel enthesitis

***Examination of the general condition, oral cavity, lymph nodes (e.g., head, neck, axils, and groin), heart and lungs, abdomen, joints, entheses, and skin. A neurological examination should be performed if relevant based on the history of previous diseases

**** Screening blood tests: Tuberculosis (QuantiFERON®-TB Gold In-Tube test or T-SPOT®.*TB* test), hepatitis B virus (HBsAg and anti-HBs), hepatitis C virus (anti-HCV), anti-nuclear-antibodies (ANA)

*****Hemoglobin, leukocytes including differentials, platelets, albumin, alanine transaminase, alkaline/basic phosphatase, and creatinine

### Registration of adverse events and adverse reactions

A risk assessment of the trial in regard to risk-adjusted management of adverse events (AE) and adverse reactions (AR) based on the Danish Medicines Agency guidelines was performed. Based on the following, the trial was assessed as a “low-risk” trial (risk level 1): 1) The Investigational Medicinal Product (IMP) is post-marketing and is not subject to stricter reporting requirements as per DKMA list available at the webpage (https://laegemiddelstyrelsen.dk/en/sideeffects/side-effects-of-medicines/medicines-with-stricter-reporting-requirements/), 2) the intervention is similar to standard clinical treatment, and 3) the intervention and the IMPs base of evidence concerning safety is robust^1^

Implementation of a risk-adjusted management of AE and AR is therefore justified.

As the trial is assessed as a “low-risk” trial, level 1 (see above), only suspected unexpected serious adverse reactions (SUSARs) are recorded and reported. Because only SUSARs are registered, it is the responsibility of the investigator to assess causality in case of AEs.

### Endpoints

#### Primary endpoint

The primary endpoint is the change from baseline to month 12 in the total inflammatory burden in the entire body (peripheral and axial joints and entheses), as assessed by WB-MRI, in patients with axSpA and PsA treated with ixekizumab.

#### Secondary endpoints

A description of key secondary and other secondary endpoints is provided in Table 3.

**Table 3:** Description of endpoints

Primary endpoint	
1.	Change from baseline to month 12 in *the total inflammatory burden in the entire body* (peripheral and axial joints and entheses), as assessed by WB-MRI, in patients with axSpA and PsA treated with ixekizumab.
**Key secondary endpoints**	
1.	Change from baseline to *months 12 and 24* in *peripheral enthesitis*, as assessed by WB-MRI, in patients with axSpA and PsA treated with ixekizumab.
2.	Change from baseline to *months 12 and 24* in *peripheral joint inflammation*, as assessed by WB-MRI, in patients with axSpA and PsA treated with ixekizumab.
3.	Change from baseline to *month 24* in *total inflammatory burden in the entire body* (peripheral and axial joints and entheses), as assessed by WB-MRI, in patients with axSpA and PsA treated with ixekizumab.
4.	Description of *patterns of structural damage in sacroiliac joints and spine* in patients with axSpA and PsA by conventional MRI methods, MRI-based synthetic CT, low-dose CT, and radiography.
5.	Description of *changes in structural damage in sacroiliac joints and spine* in patients with axSpA and PsA by conventional MRI methods, MRI-based synthetic CT, low-dose CT, and radiography, during 2 years of therapy with ixekizumab.
**Other secondary endpoints**	
1.	Change from baseline to *months 4, 12, and 24* in *axial inflammation*, as assessed by MRI, in patients with axSpA and PsA treated with ixekizumab, including separately in the posterolateral elements of the spine (facet joints, costovertebral joints, transverse processes, spinous processes, and perientheseal soft tissues) and in the vertebral bodies.
2.	Change from baseline to *months 4 and 24* in *peripheral enthesitis*, as assessed by WB-MRI, in patients with axSpA and PsA treated with ixekizumab.
3.	Change from baseline to *months 4 and 24* in *peripheral joint inflammation*, as assessed by WB-MRI, in patients with axSpA and PsA treated with ixekizumab.
4.	Change from baseline to *month 4* in *total inflammatory burden in the entire body* (peripheral and axial joints and entheses), as assessed by WB-MRI, in patients with axSpA and PsA treated with ixekizumab.
5.	*Description of differences between axSpA and PsA* inflammatory disease manifestations and their response to ixekizumab therapy at months 4, 12, and 24 (by sacroiliac joint and spine MRI and by WB-MRI).
6.	*Development of improved MRI and CT assessment methods* for axial structural damage assessment.
7.	Description of the *relationship between clinical, patient-reported, and imaging findings* at baseline and during ixekizumab therapy.

*WB-MRI* Whole body magnetic resonance imaging, *axSpA* axial spondyloarthritis, *PsA* Psoriatic arthritis, *CT* computed tomography

### Statistics

#### Sample size calculation

The primary hypothesis of this study is that ixekizumab will reduce the total inflammatory burden in the entire body of patients with axSpA and PsA, as assessed by WB-MRI. All patients in this study will be treated with ixekizumab and the primary endpoint relates to identifying changes within groups. A guiding sample size calculation was done.

A previous investigator-initiated study with 53 axSpA patients treated with a bDMARD (golimumab) using WB-MRI as an outcome measure showed that the mean change in MRI-total inflammation (MRI axial inflammation + MRI peripheral inflammation (joints and entheses)) at week 52 was 24.8 (*p* < 0.001) with a standard error of 3.6 [55].

The change in WB-MRI total inflammation is the primary endpoint in the current study. If the expected mean of the paired differences is 24.8 and the expected standard deviation is 26.2 (corresponding to a standard error of 3.6) the study would require a sample size of 12 (numbers of pairs) to achieve a power of 0.80 and a level of significance of 0.05 (two-sided).

With an expected drop-out rate of 20% at month 12 the required sample size would be 14 patients.

It is planned to include 65 patients (≥25 with each diagnosis).

Sub-group analyses in regard to the primary endpoints for all groups (axSpA vs. PsA, WB-MRI remission vs. WB-MRI non-remission and clinical responders vs. clinical non-responders) are planned. For this matter, the calculated sample size is considered appropriate to detect a statistically significant change in the total inflammatory burden in the entire body as assessed by WB-MRI at month 12 in both the overall study population and in the individual sub-groups during treatment with ixekizumab.

### Statistical analysis

The primary analysis will be the intention-to-treat (ITT) population, which comprises all included patients. An additional Per Protocol (PP) analysis will be done as a robustness analysis. The entire patient population will be analyzed for the primary and secondary endpoints. Furthermore, patients will be stratified based on their diagnosis and WB-MRI remission status (remission versus non-remission). Additionally, patients will be stratified as clinical responders versus non-responders at months 4, 12, and 24, based on the presence/absence of ASDAS low disease activity (≤2.1) for patients with axSpA and DAPSA low disease activity (≤14) for patients with PsA. The total inflammatory burden in the entire body, including both peripheral and axial joints and entheses will be assessed at multiple time points - month 0 (baseline), month 4, month 12 (the primary endpoint), and month 24. The total inflammatory burden in the entire body and the WB-MRI remission status will be determined using the sum score of WB-MRI scoring methods for peripheral (WIPE) and axial (CANDEN spine and SPARCC SIJ) inflammation. Further, patients (both the entire patient population and sub-groups stratified by diagnosis, WB-MRI remission and clinical responsiveness) will be evaluated separately for peripheral enthesitis and joint inflammation (using WIPE), and axial inflammation (using CANDEN spine and SPARCC SIJ).

The statistical analyses will include descriptive statistics for all patients (number, median/mean/range of conventional and experimental data), comparisons between groups and within groups (axSpA vs. PsA, WB-MRI remission vs. WB-MRI non-remission, and clinical responders vs. clinical non-responders).

Changes within all patients and the described sub-groups are planned to be analyzed with paired T-test (in case of normal distribution), and Wilcoxon signed rank test (in case of non-normal distribution). Comparisons between the sub-groups will be analyzed with an unpaired T-test (in case of normal distribution) or Mann-Whitney U test (non-normal distribution). Furthermore, AUC (area under the curve) values for biomarkers as well as clinical and imaging parameters will be calculated in addition to correlation analysis comparing imaging and clinical findings (in case of normal distribution) or Spearman’s test of rank correlation (non-normal distribution). Finally, standardized response mean (SRM) will be used to assess responsiveness, and an analysis of predictors will be performed by doing univariate followed by multivariable linear and logistic regression analysis. A detailed statistical analysis plan (SAP) will be made before the start of any analyses.

## Discussion

The ability of a therapy to diminish inflammation, such as peripheral and axial enthesitis, and to prevent or halt structural progression is fundamental in treating patients with axSpA and PsA. To clarify these therapeutic effects is of utmost importance, as they are essential for preventing pain and functional impairment in the patients.

In PsA, pain caused by enthesitis can be challenging to distinguish from pain resulting from central sensitization, and overlapping fibromyalgia is common in PsA, as well as in axSpA [56, 57].

Since diagnosing enthesitis through clinical methods is difficult and uncertain, clinically based outcome measures for enthesitis may not yield reliable results. WB-MRI offers the capability to objectively assess joints and entheses throughout the entire body. The DAPHNE study aims to utilize this comprehensive imaging method, along with the validated MRI WIPE scoring system, to investigate the unexplored effects of ixekizumab on enthesitis and peripheral joint inflammation.

In addition to the evaluation of inflammation in peripheral joints and entheses, axial inflammation and structural lesions will be evaluated using the CanDen MRI definitions. These definitions have recently been adopted by ASAS [58]. Inflammation in relation to each discovertebral junction, in synovial joints such as facet joints, in costo-vertebral and costo-tranversal joints and in posterior entheses at the spinous process and transverse processes can be separately assessed by this method, as can the development of structural lesions such as fat metaplasia, bone erosion and new bone formation at the individual locations. To our knowledge, no such data in ixekizumab treated patients exist.

However, radiography using the mSASSS method for structural damage assessment in the spine is still yet the only validated and recommended method for this purpose [59]. Nevertheless, low-dose CT has been shown to outperform radiography in evaluating structural damage in the spine [60–62] and although low-dose CT involves significantly less radiation than conventional CT, it still exposes patients to ionizing radiation. In contrast, synthetic CT is based on an MRI sequence, meaning that patients are not exposed to radiation. Beyond the significant advantage of eliminating radiation exposure, the MRI examination could then offer both the excellent ability to visualize inflammation of conventional MRI and the better visualization of structural damage provided by sCT.

Currently, there are no published studies on the ability of this relatively new sCT method to detect and monitor structural changes in the spine of patients with axSpA and PsA. As it is known that in radiographic axSpA, most progression in structural changes is seen in the spine [22], the ability of an imaging method to reliably detect and monitor structural changes in the spine is crucial to its use, especially in clinical trials.

Since no data exist on the objective assessment of enthesitis, inflammation of the posterolateral spinal segments, or structural progression in the spine using modern imaging during ixekizumab treatment neither in PsA nor axSpA, a longitudinal study hereof would provide important new evidence on the effectiveness of ixekizumab across these different disease domains.

In the DAPHNE study repeated MRI (including MRI-based synthetic CT) and low-dose CT scans will be performed, and the patterns of structural damage will be explored with the purpose of improving axial structural damage assessments in patients with axSpA and PsA. The study will compare the different imaging modalities and images will be evaluated with the currently available methods for structural damage assessment in the spine and the sensitivity to change will be tested. In addition, further exploration and optimization/development of new assessment methods will be done. The latter will first be possible after reading the images, as this will then reveal the limitations of the current methods and highlight areas where improvements are needed. Afterward, when the new methods are developed, it is the plan to refine and validate these, and thereafter to take the methodologies to international endorsement.

A non-randomized trial study design was chosen for several reasons: Firstly, the primary and 6 out of 7 secondary objectives are based on imaging (MR, CT, and radiography). All images will be assessed blinded to diagnosis, chronology, and clinical and other imaging findings according to internationally recognized scoring systems. Thus, observed changes will not be subject to bias and a control group is not required. Secondly, the advantages of not having a placebo-controlled trial include that the patients are not exposed to a period without active therapy. This fact minimizes patient discomfort and increases the willingness of patients to participate in the study.

In conclusion, the DAPHNE study is anticipated to significantly improve our understanding of the diverse disease manifestations in patients with axSpA and PsA, and how they respond to ixekizumab treatment. Additionally, the study will provide valuable insights into new and improved imaging examination and evaluation methods.

## Data Availability

No datasets were generated or analysed during the current study.

## Footnotes

1 https://www.ema.europa.eu/en/documents/product-information/taltz-epar-product-information_en.pdf
